# ABI5 promotes heat stress-induced chlorophyll degradation by modulating the stability of MYB44 in cucumber

**DOI:** 10.1093/hr/uhad089

**Published:** 2023-05-04

**Authors:** Weikang Liu, Guangling Chen, Mingming He, Jianqiang Wu, Wenxu Wen, Qinsheng Gu, Shirong Guo, Yu Wang, Jin Sun

**Affiliations:** College of Horticulture, Nanjing Agricultural University, Nanjing 210095, China; College of Horticulture, Nanjing Agricultural University, Nanjing 210095, China; College of Horticulture, Nanjing Agricultural University, Nanjing 210095, China; College of Horticulture, Nanjing Agricultural University, Nanjing 210095, China; College of Horticulture, Nanjing Agricultural University, Nanjing 210095, China; Zhengzhou Fruit Research Institute, Chinese Academy of Agricultural Sciences, Zhengzhou 450009, China; College of Horticulture, Nanjing Agricultural University, Nanjing 210095, China; College of Horticulture, Nanjing Agricultural University, Nanjing 210095, China; College of Horticulture, Nanjing Agricultural University, Nanjing 210095, China

## Abstract

The yellowing of leaves caused by the decomposition of chlorophyll (Chl) is a characteristic event during senescence, which can be induced by various environmental stresses. However, the molecular mechanisms of high temperature-induced Chl degradation in horticultural plants remain poorly understood. Here, we found that heat stress induced Chl degradation and the expression of *ABI5* and *MYB44* in cucumber. Silencing of *ABI5* compromised heat stress-induced Chl degradation, and the transcription of pheophytinase (*PPH*) and pheophorbide *a* oxygenase (*PAO*), two key genes in Chl catabolic pathway, but silencing of *MYB44* exhibited the opposite results. Furthermore, ABI5 interacted with MYB44 *in vitro* and *in vivo*. ABI5 positively regulated heat stress-induced Chl degradation through two pathways. ABI5 directly bound to *PPH* and *PAO* promoters to promote their expression, leading to accelerating Chl degradation. On the other hand, the interaction between ABI5 and MYB44 reduced the binding of MYB44 to *PPH* and *PAO* promoters and led to the ubiquitination-depended protein degradation of MYB44, thereby alleviating the transcription inhibitory effect of MYB44 on *PPH* and *PAO*. Taken together, our findings propose a new regulatory network for ABI5 in regulating heat stress-induced Chl degradation.

## Introduction

With global warming, high temperature occurs frequently all over the world, which has become a major stress factor for plants in recent years; it inhibits seed germination, growth and development, and accelerates leaf senescence [1, 2]. Leaf senescence contains a lot of changes in physiological, biochemical, and molecular levels, such as reducing chlorophyll (Chl) contents and photosynthetic capacity, proteins and nucleic acids degradation, and nutrient remobilization [3–5]. Among these, Chl degradation is an obvious symptom of leaf senescence [6]. Generally, leaf premature senescence results in decrease the yield of crops. Therefore, it is necessary to understand the mechanisms of leaf senescence.

**Figure 1:**
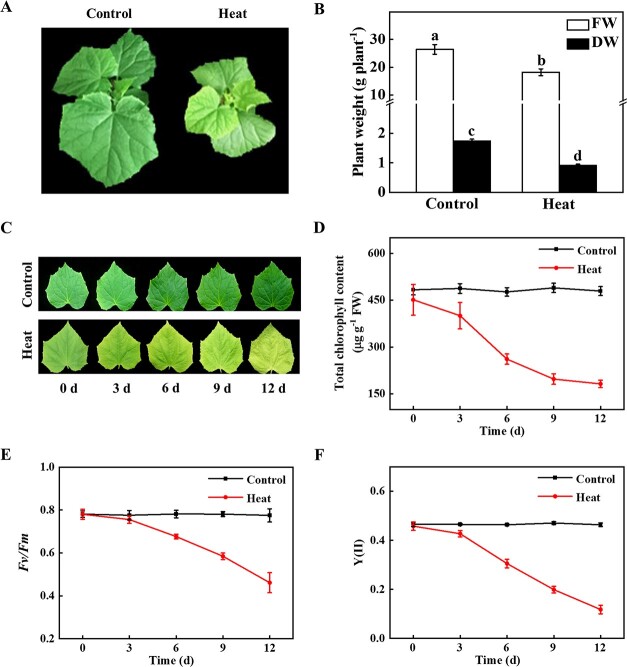
Heat stress induced chlorophyll degradation in cucumber leaves. **A** The phenotype of cucumber seedlings under heat stress for 9 d. **B** Fresh weight (FW) and dry weight (DW) of cucumber seedlings. **C** The phenotype of the second leaves from 3-week-old cucumber plants after heat treatment for 0, 3, 6, 9, and 12 d. **D** Total chlorophyll contents in cucumber leaves shown in **C**. **E***Fv/Fm* of cucumber. **F** Y(II) of cucumber. The FW, DW, *Fv/Fm*, and Y(II) were detected at 12 d of heat treatment. The results represent the mean ± SD (*n* = 3). Different letters on each column indicated significant differences at *P* < 0.05.

During the past two decades, the key genes involved in Chl breakdown were identified in Arabidopsis (*Arabidopsis thaliana*), including non-yellow coloring 1 (*NYC1*), NYC1-like (*NOL*), hydroxymethyl chl *a* reductase (*HCAR*), stay-green (*SGR*), pheophytinase (*PPH*), pheophorbide *a* oxygenase (*PAO*), and red Chl catabolite reductase (*RCCR*) [4, 7]. The catabolism of Chl is regulated by phytohormones, sugar, and light signals [6–8]. Absisic acid (ABA), jasmonic acid, salicylic acid (SA), ethylene (ET), and strigolactone promote Chl degradation, while melatonin suppresses leaf degreening through activating antioxidant pathway to scavenge overaccumulated reactive oxygen species (ROS) and reducing the expression of Chl catabolic and ABA biosynthesis genes [9–16]. ABA mediates leaf senescence either through ET-dependent or ET-independent pathway via activating SnRK2s to phosphorylate ABFs [15]. ET and SA coordinately accelerate leaf senescence [13, 14]. Therefore, phytohormones regulate Chl catabolic synergistically or antagonistically.

Except for phytohormones, transcription factors (TFs) also mediate Chl catabolism by directly regulating the expression of genes related to Chl degradation [4, 17]. Chinese flowering cabbage WRKY65 is induced during Chl breakdown, and directly binds to *NYC1* and *SGR1* promoters to activate their expression [18]. Furthermore, NAC and MYC TFs not only directly activate the transcription of Chl degradation genes, but also elevate ABA content to promote Chl degradation [19–23]. ABFs act as the downstream of ABA signaling to directly trigger the transcription of Chl catabolic genes [24]. ABI5 involves in ABA-mediated Chl catabolism and abiotic stress adaptation [3, 25, 26]. MYB TFs, the most widely distributed TF family in plants, play important roles in abiotic and biotic stresses response [27, 28]. Overexpression of *MYB102* suppresses the Chl degradation through down-regulating the expression of senescence-associated genes and ABA signaling [29]. Similarly, MYB44 is a R2R3-MYB TF, and its overexpression plants delay leaf senescence, but *myb44* mutants display more rapid Chl degradation and senescence, indicating that MYB44 negatively regulates Chl catabolic in Arabidopsis [30]. However, the molecular mechanism of MYB44-mediated Chl degradation remains elusive. Interestingly, MYB44 interaction with RCAR1/PYL9, an ABA receptor, increases the phosphatase activity of ABI1 [31]. However, whether MYB44 is involved in regulating ABA-mediated Chl degradation is largely unknown.

Here, we found that heat stress induced *ABI5* and *MYB44* expression. ABI5 positively regulated, but MYB44 negatively regulated heat stress-induced Chl degradation in cucumber (*Cucumis sativus* L.). On the one hand, ABI5 promoted the degradation of Chl through directly up-regulating *PPH* and *PAO* expression. On the other hand, the interaction between ABI5 and MYB44 reduced the binding of MYB44 to *PPH* and *PAO* promoters and led to the ubiquitination-depended protein degradation of MYB44, leading to faster Chl degradation. Our work provides a novel insight for transcriptional regulation of Chl degradation.

**Figure 2:**
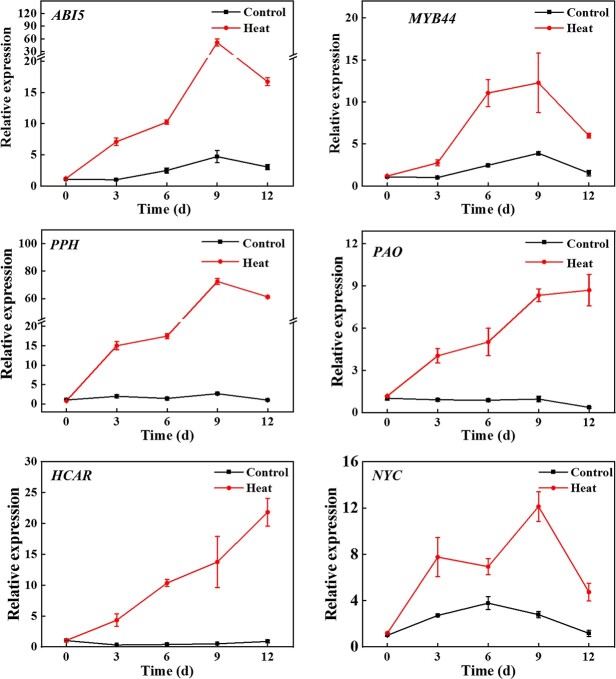
Heat stress enhanced the transcription of *ABI5*, *MYB44*, and chlorophyll catabolic genes. qPCR analysis of the transcription level of *ABI5*, *MYB44*, and chlorophyll catabolic genes in cucumber leaves under heat stress. The results represent the mean ± SD (*n* = 3).

## Results

### Heat stress accelerates Chl degradation in cucumber

Heat stress (42/32°C, day/night) significantly inhibited plant growth, as indicated by lower fresh weight (FW) and dry weight (DW) compared with the control plants (Fig. 1A and B). The color and Chl content of cucumber plants grown under optimum temperature had no significant difference (Fig. 1C and D). However, heat stress induced leaf chlorosis and the Chl content gradually decreased with the treatment elongation (Fig. 1C and D). Under normal growth conditions, the maximum quantum yield of photosystem II (*Fv/Fm*) and effective quantum yield of photosystem II [Y(II)] of cucumber seedlings was stable and had no significant change (Fig. 1E and F). With the elongation of heat treatment, *Fv/Fm* and Y(II) values gradually decreased, they decreased by 41.02% and 73.9%, respectively, at 12 d compared with the control plants (Fig. 1E and F). Thus, heat stress negatively regulated plant growth and accelerated Chl degradation in cucumber.

### Heat stress enhances the expression of *ABI5*, *MYB44*, and Chl catabolic genes

To test the function of ABI5 and MYB44 in heat-induced Chl degradation, we analysed their expression level during heat stress using qPCR. The expression of *ABI5* and *MYB44* was induced by heat stress in cucumber and reached peak at 9 d (Fig. 2). Consideration the vital role of Chl catabolic genes during Chl degradation, their expression patterns under heat stress were analysed. Strikingly, the expression level of *PPH*, *PAO*, *HCAR*, and *NYC* was drastically up-regulated under heat stress (Fig. 2). However, other homologs of *PPH* and *PAO* only slightly changed under heat stress (Fig. S1, see online supplementary material). Furthermore, the activity of PPH and PAO was constant under the normal growth conditions, but significantly increased under heat stress (Fig. S2, see online supplementary material). These results suggested that heat stress elevated the transcription of Chl catabolic genes, increased their activities to promote Chl degradation.

### ABI5 directly binds to *PPH* and *PAO* promoters

**Figure 3:**
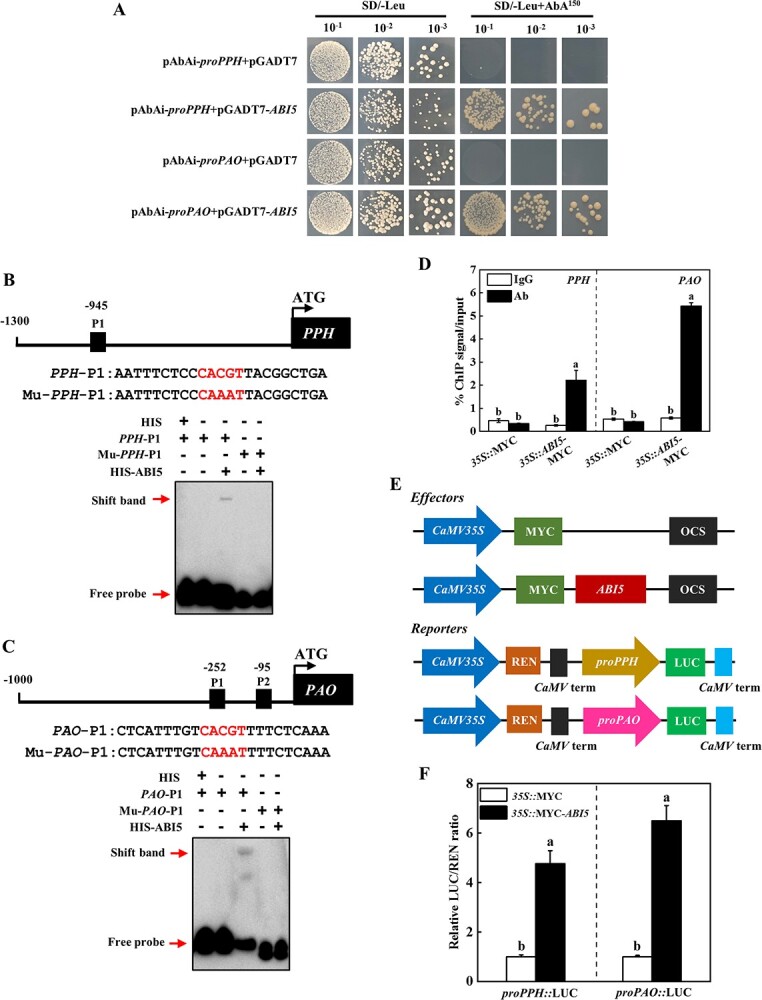
ABI5 directly activates the transcription of *PPH* and *PAO*. **A** Yeast one-hybrid assay indicating ABI5 binding to *PPH* and *PAO* promoters. **B** Electrophoretic mobility shift assays (EMSA) showing ABI5 binding to *PPH* promoter. Schematic representations of *PPH* promoter and the probe used for EMSA. **C** EMSA showing ABI5 binding to *PAO* promoter. Schematic representations of *PAO* promoter and the probe used for EMSA. The ABI5 binding sites in the probes are shown in red in (**B** and **C**). **D** ChIP-qPCR analysis of ABI5 binding to the promoters of *PPH* and *PAO*. The ChIP results represent percentages of input DNA. **E** Effector and reporter’s schematic diagram. **F** Luciferase assay analysis the effects of *ABI5* on the expression of *PPH* and *PAO*. The results represent the mean ± SD (*n* = 3). Different letters on each column indicated significant differences at *P* < 0.05.

To test whether ABI5 regulated Chl catabolic genes, we first used yeast one-hybrid (Y1H) assays to analyse the binding activity of ABI5 to Chl catabolic genes promoters. Only the bait vector containing the promoter sequence of *PPH* or *PAO* grew on solid medium with 150 ng mL^−1^ aureobasidin A (AbA) when transformed with pGADT7-*ABI5* (Fig. 3A). Sequence analysis revealed that there were one and two ABRE elements (ACGT) in the promoters of *PPH* and *PAO*, respectively (Fig. 3B and C). We used an electrophoretic mobility shift assay (EMSA) to further test whether ABI5 protein directly bound to *PAO* and *PPH* promoters. The DNA fragment harboring the ABRE elements or the mutant elements labeled with 3′ biotin was used as the probe (Fig. 3B and C). EMSA results observed that ABI5 bound to the labeled probe of *PPH*-P1 and *PAO*-P1, while no shift bands were detected when added the mutant probes or *PAO*-P2 probe (Fig. 3B and C; Fig. S3, see online supplementary material). In addition, the biotin-labeled probes incubated with the negative control of HIS did not produce a mobility shift (Fig. 3B and C). Furthermore, we performed chromatin immunoprecipitation coupled with qPCR (ChIP-qPCR) assays to detect whether ABI5 bound to *PPH* and *PAO* promoters *in vivo*. The cotyledons of transiently expressed MYC-tagged ABI5 were collected for ChIP-qPCR assays, and found that the promoter sequences of *PPH* and *PAO* were precipitated from the cotyledons injection with *35S::ABI5*-MYC using an anti-MYC antibody (Fig. 3D; Fig. S4A, see online supplementary material). However, no sequences were precipitated from the control cotyledons (Fig. 3D). These results suggested that ABI5 could specifically bind to the promoters of *PPH* and *PAO*.

To determine whether ABI5 directly regulates the transcription of *PPH* and *PAO*, the dual luciferase assays were used to detect the activity of ABI5 in the activation of *PPH* and *PAO* expression. For the reporter construct, the promoter of *PPH* or *PAO* was fused with the LUC reporter, respectively (Fig. 3E). Co-expression of *35S::ABI5*-MYC and *proPPH::*LUC, *35S::ABI5*-MYC and *proPAO::*LUC increased the relative LUC/REN ratio, which was 4.8–6.5-fold of the control leaves (Fig. 3F). Thus, ABI5 directly bound to *PPH* and *PAO* promoters to activate their transcription.

### MYB44 directly binds to *PPH* and *PAO* promoters

**Figure 4:**
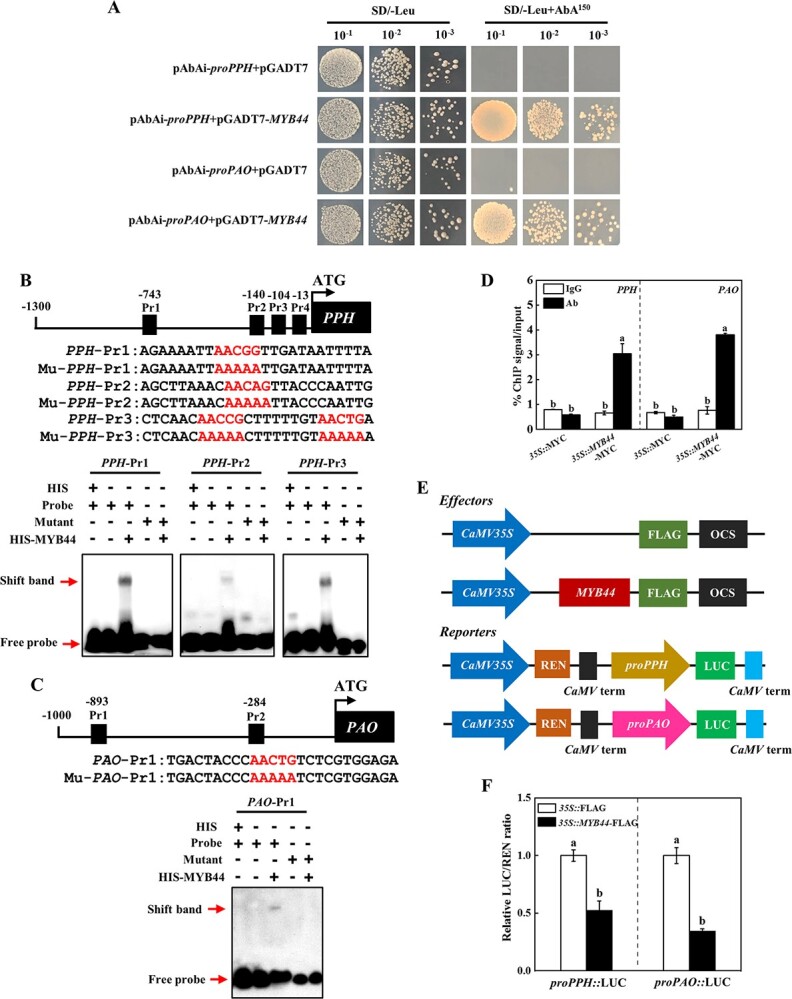
MYB44 directly inhibits the transcription of *PPH* and *PAO*. **A** Yeast one-hybrid assay indicating MYB44 binding to *PPH* and *PAO* promoters. **B** EMSA showing MYB44 binding to *PPH* promoter. Schematic representations of *PPH* promoter and the probe used for EMSA. **C** EMSA showing MYB44 binding to *PAO* promoter. Schematic representations of *PAO* promoter and the probe used for EMSA. The MYB44 binding sites in the probes are shown in red in **B** and **C**. **D** ChIP-qPCR analysis of MYB44 binding to the promoters of *PPH* and *PAO*. The ChIP results represent percentages of input DNA. **E** Effector and reporter’s schematic diagram. **F** Luciferase assay analysis the effects of *MYB44* on the expression of *PPH* and *PAO*. The results represent the mean ± SD (*n* = 3). Different letters on each column indicated significant differences at *P* < 0.05.

Y1H assays showed that MYB44 could bind to *PPH* or *PAO* promoter (Fig. 4A). Interestingly, the promoters of *PPH* and *PAO* contained four and two MYB binding sites (MBS element, AACNG), respectively (Fig. 4B and C). We used an EMSA to further investigate whether MYB44 protein directly bound to the promoters of *PPH* and *PAO*. EMSA results showed that MYB44 bound to the labeled probe of *PPH*-Pr1, *PPH*-Pr2, *PPH*-Pr3, and *PAO*-Pr1, but failed to bind to these mutant probes, *PPH*-Pr4 or *PAO*-Pr2 (Fig. 4B and C; Fig. S5, see online supplementary material). However, the biotin-labeled probes incubated with only HIS did not produce a mobility shift (Fig. 4B and C). These results indicated that MYB44 specifically bound to the MBS elements of *PPH* and *PAO* promoters *in vitro*. Furthermore, ChIP-qPCR results found that MYB44 bound to the promoter of *PPH* and *PAO in vivo* (Fig. 4D; Fig. S4B, see online supplementary material). However, the LUC/REN ratio significantly decreased when co-inoculated with the *35S::MYB44*-FLAG effector and reporters (Fig. 4E and F). Yeast two hybrid assay showed that MYB44 was a transcription repressor (Fig. S6, see online supplementary material). Therefore, these results suggested that MYB44 directly bound to *PPH* and *PAO* promotors, and inhibited their transcription.

### ABI5 and MYB44 is involved in heat-induced Chl degradation

**Figure 5:**
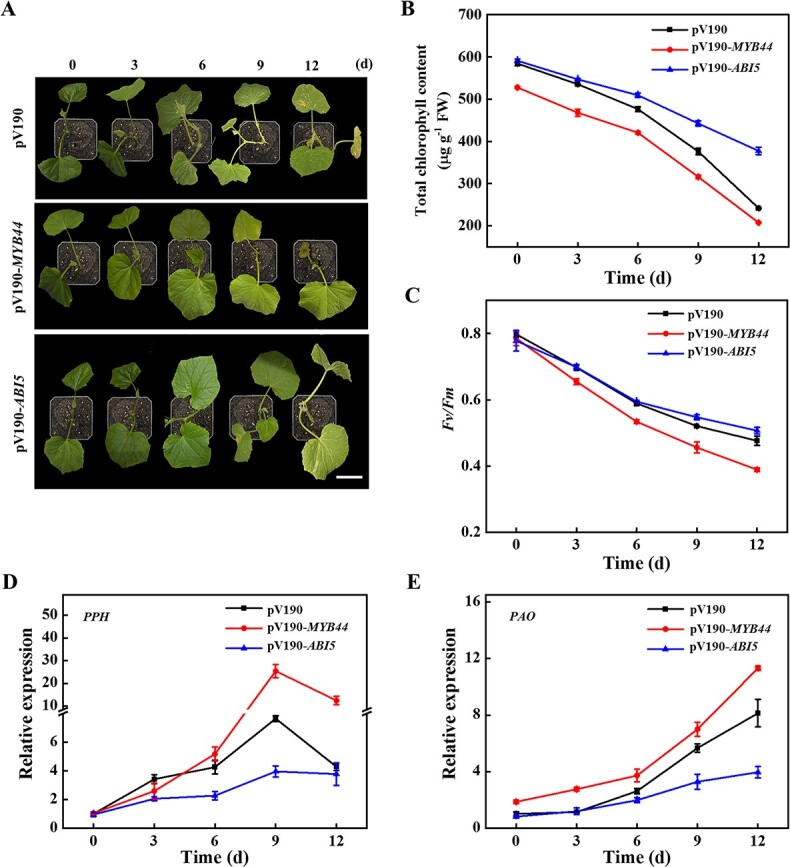
Effects of *ABI5* and *MYB44* on heat-induced chlorophyll degradation. **A** The phenotype of *ABI5* and *MYB44* silenced plants under heat stress. Bar: 5 cm. **B** Total chlorophyll contents in *ABI5* and *MYB44* silenced plants. **C***Fv/Fm* in *ABI5* and *MYB44* silenced plants. **D** and **E** qPCR analysis the expression of *PPH* and *PAO* in the leaves of *ABI5* and *MYB44* silenced plants. The results represent the mean ± SD (*n* = 3).

To test the function of ABI5 and MYB44 in heat stress-induced Chl degradation, we silenced *ABI5* and *MYB44* genes using the method of virus-induced gene silencing (VIGS) with a cucumber green mottle mosaic virus vector (pV190) that is highly efficiency to knockdown gene expression in cucumber [32]. Phytoene desaturase (*PDS*)-silenced plants displayed the photobleaching phenotypes (Fig. S7A, see online supplementary material). Furthermore, the transcript level of *ABI5* in pV190-*ABI5* or *MYB44* in pV190-*MYB44* was significantly lower than that detected in pV190 plants (Fig. S7B, see online supplementary material). Although heat stress induced Chl degradation and *Fv/Fm* decline in all of the plants, the Chl content and the value of *Fv/Fm* in pV190-*MYB44* plants were lower than those of pV190 plants, and those in pV190-*ABI5* plants showed higher levels comparison to pV190 plants (Fig. 5A–C). Moreover, the transcription levels of *PPH* and *PAO* began to increase after 3 d of heat stress, but the up-regulation level was the most obvious in pV190-*MYB44* (Fig. 5D and E). The transcription level of *PPH* in pV190-*MYB44* plants reached the maximum at 9 d of heat stress, which was 3.3-fold that in pV190 plants (Fig. 5D). The transcription level of *PAO* in pV190-*MYB44* gradually increased with the elongation of heat stress, which was 23.5% higher than that in pV190 plants at 12 d (Fig. 5E). However, the expression levels of *PPH* and *PAO* in pV190-*ABI5* were lower than that in pV190 (Fig. 5D and E). These above results indicated that ABI5 and MYB44 were both involved in regulating the expression of *PPH* and *PAO* to mediate Chl degradation.

### ABI5 interacts with MYB44 protein

**Figure 6:**
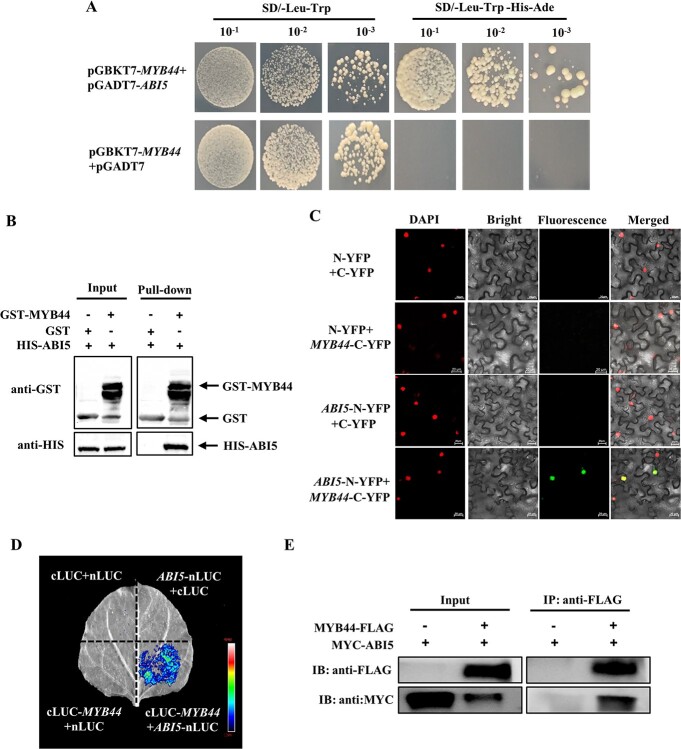
ABI5 interacts with MYB44. **A** Y2H showing ABI5 interaction with MYB44. **B** Pull-down assay detection ABI5 interaction with MYB44. The recombinant HIS-ABI5 was incubated with GST or GST-MYB44. Proteins were immunoprecipitated by GST-tag purification resins and detected with an anti-GST and anti-HIS antibody, respectively. **C** BiFC analysis the interaction between ABI5 and MYB44 proteins in the nuclei of *Nicotiana benthamiana* leaves. DAPI was used to stain the nuclei. Bars: 20 μm. **D** Firefly luciferase complementation assay analysis ABI5 interaction with MYB44. **E** Co-immunoprecipitation (Co-IP) assays indicating ABI5 interaction with MYB44 *in vivo*.

To investigate the potential mechanism of ABI5 and MYB44 in Chl degradation, we detected whether ABI5 and MYB44 interacted with each other. Yeast two-hybrid (Y2H) assays demonstrated that ABI5 interacted with MYB44 (Fig. 6A). Furthermore, pull-down assays found that HIS-ABI5 was pull-down by GST-MYB44, while failed using the control GST protein (Fig. 6B). To further verify ABI5 interaction with MYB44, *ABI5*-N-YFP and *MYB44*-C-YFP were transformed into tobacco (*Nicotiana benthamiana*) leaves. The fluorescence signals were detected only when *ABI5*-N-YFP and *MYB44*-C-YFP were simultaneously transformed (Fig. 6C), and the fluorescence signals located in the nuclear, indicating that ABI5 interacted with MYB44 in the nucleus. In addition, luciferase activity was measured in the leaves co-transformed with ABI5 and MYB44 (Fig. 6D). Co-immunoprecipitation (Co-IP) results illustrated that ABI5 interacted with MYB44 *in vivo* (Fig. 6E). Therefore, the results demonstrated that ABI5 directly interacted with MYB44.

### ABI5 inhibits the binding of MYB44 to *PPH* and *PAO* promoter

**Figure 7:**
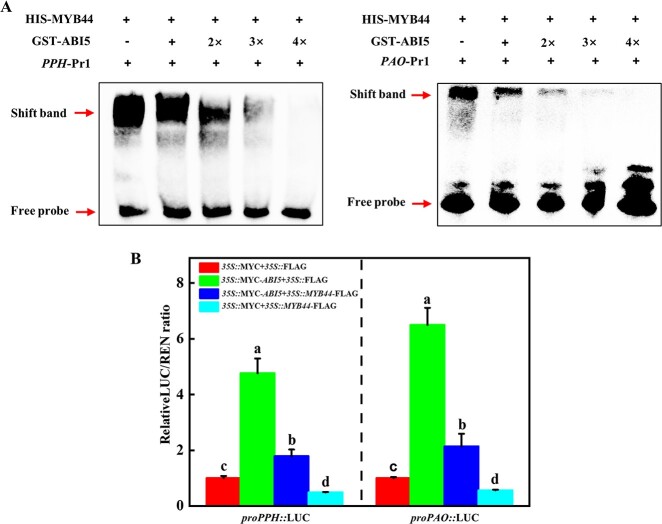
ABI5 interferes the binding of MYB44 *PPH* and *PAO* promoters. **A** EMSA indicated that enhancing the amount of GST-ABI5 inhibited HIS-MYB44 binding to *PPH* and *PAO* promoters. **B** Luciferase assay indicating transient co-overexpression of ABI5 and MYB44 repression the transcriptional inhibitory effect of MYB44 on *PPH* and *PAO*. The results represent the mean ± SD (*n* = 3). Different letters on each column indicated significant differences at *P* < 0.05.

Due to the interaction of ABI5 and MYB44, and the expression patterns of *PPH* and *PAO* in *ABI5* and *MYB44* silencing plants were the opposite under heat stress, we proposed that ABI5 interacted with MYB44 to inhibit its binding to *PPH* and *PAO* promoters*.* To test this hypothesis, we performed EMSA assays to detect the binding activity of MYB44 to the probes of *PPH*-Pr1 and *PAO-*Pr1 in the presence of ABI5*.* The shift bands were detected with HIS-MYB44 (Fig. 7A). However, the binding intensities of MYB44 gradually decreased with the increasing amounts of GST-ABI5 (Fig. 7A), suggesting that ABI5 inhibited the binding of MYB44 to *PPH* and *PAO* promoters. In contrast, the binding intensities of ABI5 to *PPH* and *PAO* promoters were not affected in the presence of MYB44 (Fig. S8A, see online supplementary material). To further confirm these results, dual luciferase assays were performed to test the expression of *PPH* and *PAO* in the presence of ABI5 and MYB44. As shown in Fig. S8B and C (see online supplementary material), the proteins of ABI5 and MYB44 were detected in the tobacco leaves injection of *35S::*MYC*-ABI5* and *35S::MYB44*-FLAG. Furthermore, the relative LUC/REN ratio significantly decreased only inoculation of MYB44, but drastically increased when inoculated with ABI5 and MYB44 (Fig. 7B). Thus, ABI5 interacted with MYB44 to interfere MYB44 binding activity to its target genes.

### ABI5 mediates the ubiquitination and degradation of MYB44

**Figure 8:**
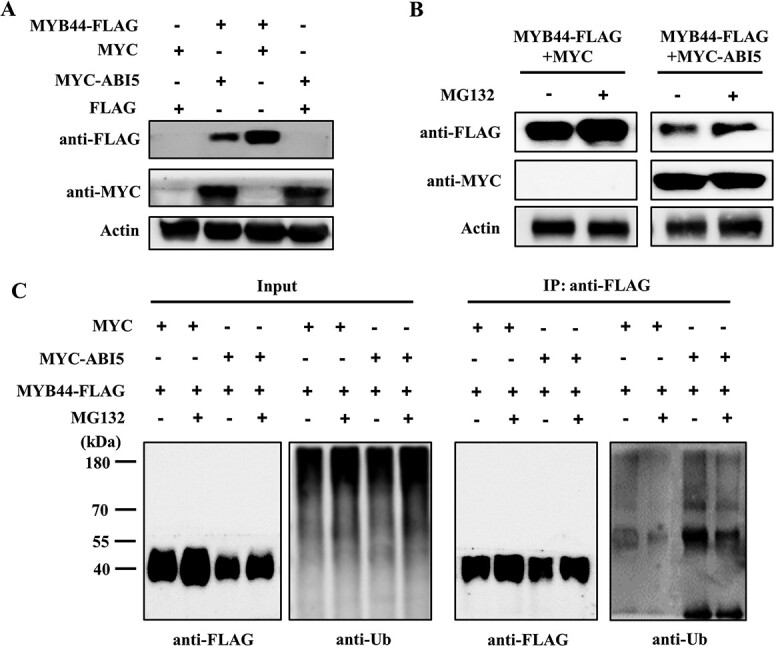
ABI5 mediates the ubiquitination and degradation of MYB44. **A** ABI5 promoted the degradation of MYB44. **B** Treatment with MG132 increased the abundance of MYB44-FLAG. *Nicotiana benthamiana* leaves were treated with MG132 after *Agrobacterium*-transformation for 48 h and the samples were collected after MG132 treatment for 2 h. **C** ABI5 promoted the level of ubiquitinated MYB44.

To test the role of ABI5 on the degradation of MYB44, we analysed the abundance of MYB44 in the present or absent of ABI5. The abundance of MYB44 reduced in the presence of ABI5 (Fig. 8A). To determine whether 26S proteasomes mediate the degradation of MYB44, protein levels were measured in tobacco leaves infiltrated with MG132, which is used to inhibit the activity of 26S proteasomes. As shown in Fig. 8B, treatment with MG132 efficiently attenuated the breakdown of MYB44, suggesting that the 26S proteasome pathway mediated the degradation of MYB44. We speculated that ABI5 might regulate the stability of MYB44 through modulating its ubiquitination degradation. To confirm this supposition, an immunoprecipitation (IP) assay was performed. In the IP assay, the MYB44-FLAG protein was enriched with the anti-FLAG magnetic beads, and subjected into immunoblotting analysis using an anti-FLAG or anti-Ub antibody. The ubiquitinated form of MYB44-FLAG was detected by anti-Ub antibody (Fig. 8C). The ubiquitination level of MYB44-FLAG was more evident in the presence of ABI5, but decreased when treated with MG132 (Fig. 8C). These results suggested that MYB44 stability was influenced by ABI5 through 26S proteasome-dependent ubiquitin pathway.

## Discussion

It has been demonstrated that Chl degradation is finely regulated by numerous genes [6, 11]. *PPH* and *PAO* are two key genes in Chl degradation [4, 33]. Studies have shown that TFs, such as NAC, ERF, and SOC, bind to the promoters of *PPH* or *PAO* and accelerate leaf degreening [4, 23]. Although the transcription regulation mechanism of Chl degradation in Arabidopsis has been systematically studied, their roles are largely unknown in horticultural plants. Here, we found that heat stress induced leaf yellowing, the expression of *ABI5*, *MYB44*, *PPH*, and *PAO* in cucumber. ABI5 directly bound to *PPH* and *PAO* promoters to trigger their expression, while MYB44 inhibited their expression. Furthermore, ABI5 interacted with MYB44 to mediate its degradation through 26S proteasome pathway, therefore promoting Chl degradation.

ABI5 plays a key role in ABA-mediated abiotic stress adaptation, such as heat, cold, and drought stresses [25, 34]. In addition, it is also involved in regulation seed germination [35]. *ABI5* gene knockout mutants have higher germination rates at high temperatures, suggesting that ABI5 plays critical roles in heat-induced seed dormancy [36, 37]. FCA, a RNA-binding protein, interacts with ABI5 to enhance heat stress tolerance through modulating the expression of genes related to antioxidants [38]. Furthermore, ABI5 is a key regulator in leaf senescence. Overexpression of *ABI5* in potato (*Solanum tuberosum*) promotes dark-induced leaf degreening and senescence, significantly declines Chl content in comparison to wild-type (WT) plants [34]. ABI5 directly binds to the promoter of Chl metabolism genes, and positively promotes Chl degradation [3]. Interestingly, *ABI5* gene expression was significantly up-regulated under high temperature in cucumber (Fig. 2). Y1H, EMSA, ChIP-qPCR, and dual luciferase assays demonstrated that ABI5 directly bound to the promoters of *PPH* and *PAO* genes (Fig. 3). There were two ACGT motif in the promoter of *PAO*, and EMSA assay showed that ABI5 only bound the probe of *PAO*-P1 (Fig. 3C). Previous studies also found that ABI5 has different binding efficiency with different flanking of ACGT motif and the binding efficiency is the best in the sequence containing the consensus G-box motif CACGTG [39, 40], indicating that base sequences on both sides of the ACTG motif influence ABI5 binding ability. The core sequence in the probe of *PAO*-P1 was CACGT, while in the probe of *PAO*-P2 was AACGT (Fig. 3C; Fig. S3, see online supplementary material), which might mediate the difference binding activity of ABI5. Furthermore, silencing of *ABI5* delayed heat stress-induced leaf yellowing, and suppressed the expression level of *PPH* and *PAO* (Fig. 5). These results indicated that ABI5 positively regulated Chl degradation induced by high temperature in cucumber. Recently, it has been demonstrated that WRKY40, bZIP44, and ZAT10 interacted with ABI5 to strengthen its transcriptional activity on *NYC1* and *NYE1* in apple (*Malus domestica*) [26, 41]. However, BBX22 inhibits the transcriptional activity of ABI5 on these Chl catabolic genes by direct protein interaction [26]. Thus, ABI5 acts as the core TF to interact with the positive or negative factors to modulate Chl degradation.

MYB44 plays important regulatory roles in abiotic and biological stresses response [30, 42–44]. MYB44 of yellowhorn (*Xanthoceras sorbifolium*) improves combined drought and heat resistance through regulation stomatal closure and ROS homeostasis [45]. Similarly, heat stress enhanced the transcript level of *MYB44* in cucumber, and silencing of *MYB44* dramatically decreased heat tolerance and accelerated leaf yellowing (Fig. 4; Fig.S1, see online supplementary material). Several studies showed that MYB TFs negatively regulate Chl breakdown [29, 30]. Arabidopsis *myb44* and *myb77* mutants exhibit early leaf senescence and accelerate Chl degradation relative to WT plants [30]. Chromatin immunoprecipitation results showed that rice MYB102 (a homologous gene of *MYB44* in Arabidopsis) binds to the *cis*-acting element of AACNG [29]. Interestingly, the promoters of cucumber *PPH* and *PAO* contained this *cis*-acting element (Fig. 4B and C). The probe of *PPH*-Pr1, *PPH*-Pr2, *PPH*-Pr3, and *PAO*-Pr1 had the typical MBS element C/TAACNG, while the probe of *PPH*-Pr4 and *PAO*-Pr2 only contained the core sequences of AACNG (Fig. 4B and C; Fig. S5, see online supplementary material). Indeed, MYB44 only bound to the probe of *PPH*-Pr1, *PPH*-Pr2, *PPH*-Pr3, and *PAO*-Pr1 to suppress the expression of *PPH* and *PAO* in cucumber (Fig. 4). Interestingly, *MYB44* silencing plants were more sensitive to high temperature, along with lower Chl content and up-regulated the expression of Chl degradation genes (Fig. 5). In accord with previous results, this work indicated that cucumber MYB44 negatively regulated Chl degradation induced by heat stress. It has been shown that MYB44 mediates ABA and other stress signaling pathways [31, 46]. Exogenous ABA rapidly induces the transcription of *MYB44*, and *MYB44* overexpression plants are more sensitive to ABA and increase ABA-mediated stomatal closure to enhance abiotic stresses [42], indicating that MYB44 might play critical roles in the downstream of ABA. Intriguingly, we found that ABI5 interacted with MYB44 to promote its degradation via 26S proteasome pathway, resulting in attenuation its transcription inhibitory activity for *PPH* and *PAO* (Figs 7 and 8). Previous studies showed that the RING-type E3 ligase MIEL1 interacts with and ubiquitinates MYB30 and MYB96 to facilitate their degradation [47, 48]. Furthermore, MIEL1 also interacts with ABI5 to modulate seed germination in Arabidopsis [49]. However, whether MIEL1 mediates ABI5-induced the degradation of MYB44 in cucumber requires further investigation. Although MYB44 had no influence on ABI5 protein stability and the binding activity of ABI5 to *PPH* and *PAO* promoters (Fig. 8A and B; Fig. S8A, see online supplementary material), the expression of *PPH* and *PAO* was dramatically suppressed when injected with ABI5 and MYB44 compared with only inoculation of ABI5 (Fig. 7C). It is possible that MYB44 interaction with ABI5 affected its transcriptional activation activity or remained a higher level of MYB44 binding to the promoter of *PPH* and *PAO* to suppress their expression, but this hypothesis needs further verification.

**Figure 9:**
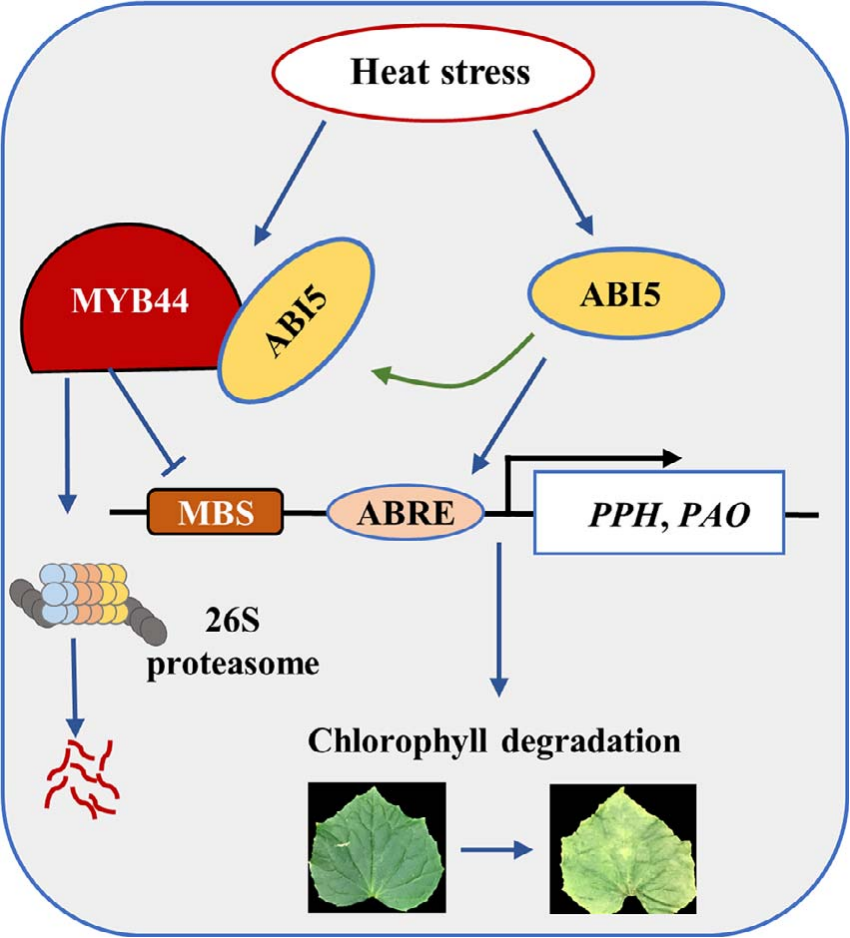
Proposed model depiction the key role of ABI5 in heat-induced chlorophyll (Chl) degradation. ABI5 is a positive regulator in heat-induced Chl degradation. On the one hand, ABI5 directly induces the transcription of *PPH* and *PAO* to promote Chl degradation. On the other hand, MYB44 is a negative regulator in heat-induced Chl degradation. ABI5 interaction with MYB44 reduces the binding of MYB44 to *PPH* and *PAO* promoters and leads to the ubiquitination-depended protein degradation of MYB44, thereby enhancing the transcription of *PPH* and *PAO* to further promote Chl degradation.

In summary, our study provides a new insight for the role of ABI5 and MYB44 in heat stress-induced cucumber Chl degradation (Fig. 9). ABI5 positively regulated cucumber Chl degradation through two pathways. ABI5 up-regulated the expression of *PPH* and *PAO* to promote Chl degradation. On the other hand, ABI5 interacted with MYB44 to inhibit MYB44 binding to the promoter of *PPH* and *PAO* and to induce MYB44 degradation via 26S proteasome, resulting in alleviating the inhibitory effect of MYB44 on *PPH* and *PAO* genes.

## Materials and methods

### Plant material and treatment

The germinated cucumber (Jinchun No. 2) seeds were sown in the plastic pots (10 cm × 7 cm × 8 cm), which were filled with the seedling substrate (peat: vermiculite = 2:1, v:v). After sowing, the seeds were placed in the growth chamber, where the temperature was 25°C/18°C (day/night), the relative air humidity was maintained at 75%–80%, the photosynthetic photon flux density was 300 μmol m^−2^ s^−1^ with 14/10 h (light/dark) cycle.

For heat stress, the cucumber seedlings with two true leaves were treated at 42/32°C (day/night). The leaves were harvested at 0, 3, 6, 9, and 12 d for growth parameters, Chl content, Chl fluorescence, and gene expression analysis.

### Total RNA extraction and gene expression analysis

Total RNAs were extracted from the leaves of cucumber with a total RNA isolation kit (Tiangen, DP419), and reverse transcribed into cDNA using a reverse transcription kit (Vazyme, R223–01). The qPCR assays was performed according to the method of Liu *et al.* [50]. The cucumber *actin* and *ubiquitin* genes were used as internal controls. The primers used in the experiments are shown in Table S1 (see online supplementary material).

### Determination of growth and Chl fluorescence parameters

After heat stress for 12 d, three cucumber plants were collected and washed with ddH_2_O. Then, the remaining water was sucked up, and the FW was detected using an electronic balance (Huazhi, Putian, China). The plant was enclosed in an envelope and placed in an oven (DHG-9030A, Shanghaiyiheng, China) for 30 min, which was set at 105°C. Then, the plant was dried at 75°C for 2 d to measure the DW. For measurement of the Chl fluorescence parameters, cucumber plants at 12 d of heat stress were placed in the dark for 30 min and were detected using a portable fluorimeter (PAM-2100, Walz, Effeltrich, Germany) [51].

### Chl content measurement

The second leaves (0.2 g) of cucumber plants were shredded and soaked in a tube, which contained 25 mL ethanol and was placed in the dark for 2 d. Then, the concentrations of Chl were determined at 665 and 649 nm, as described previously [52].

### VIGS vectors construction and **A*grobacterium*-mediated virus infection

For silencing of *ABI5*, *MYB44*, and *PDS* genes, the sequences of *ABI5* and *MYB44* were amplified with primers (Table S2, see online supplementary material) and inserted into the pV190 vector. VIGS assays were performed as per the method described by Zhang *et al.* [53]. The transcript levels of *ABI5* and *MYB44* in the leaves of VIGS plants were measured. The silenced plants, which displayed approximately 30 to 45% expression levels of the pV190 plants, were used for heat stress.

### EMSA assays

The biotin-labeled probes (ABI5-probe and MYB44-probe) were synthesized by Sangon Biotechnology Company, Ltd (Shanghai, China). The HIS-MYB44 and HIS-ABI5 fusion proteins were obtained by inducing *Escherichia coli* BL21 (DE3) with 0.1 mM IPTG. The probes were separated after the probes and fusion proteins incubation at 24°C for 30 min as previously described [54].

### ChIP-qPCR assays

The full-length CDS of *ABI5* or *MYB44* were inserted into the pCAMBIA1300 vector, and transformed into *Agrobacterium tumefaciens* strain GV3101 (Tolobio) to inject into the cotyledon of 8-d old cucumber seedlings according to the previously described method [55]. Cotyledons of transient expressing MYC-tagged ABI5 or MYB44 were used for ChIP assays. After injection for 2 d, the leaves (5 g) were collected for ChIP-qPCR analysis using the EpiQuik Plant ChIP Kit (Epigentek, P-2014) with an anti-MYC antibody (MBL, M192–3). The negative control was performed with an anti-mouse IgG (ZSGB-BIO, ZB-2305). ChIP-qPCR was performed using the primers for *PPH* and *PAO* promoters (Table S3, see online supplementary material).

### Dual luciferase assays

The full-length CDS of *ABI5* and *MYB44* were cloned into the pFGC5941-MYC and pFGC1008-FLAG, respectively. The promoter fragment of *PPH* or *PAO* was inserted into the pGreen II0800-LUC vector. The recombinant vector was inoculated into tobacco leaves as previously described [56]. Empty plasmid was used as control. The luciferase activities were measured using a Dual-Luciferase® Reporter Assay System (Promega, E1910).

### Y1H assays

The Y1H assays were performed as previously described [57]. *PPH* and *PAO* promoter sequences cloned into the pAbAi vector. The CDS of *ABI5* and *MYB44* were cloned with primers (Table S2, see online supplementary material) and inserted into the pGADT7 vector. The constructed pAbAi vectors were linearized by *Bst*B I and transferred into Y1H yeast, receptively. The transformed yeast was suspended with 100 μL 0.9% NaCl and coated on SD/-Ura medium. Single colonies were selected for PCR identification using Matchmaker Insert Check PCR Mix 1 (Clontech, 630 496). The pGADT7-*ABI5* and pGADT7-*MYB44* recombinant vectors were transformed into the verified yeast cells, which were grown on the SD/−Leu solid medium with 150 ng mL^−1^ AbA.

### Y2H assays

The CDS of *MYB44* were amplified with specific primers (Table S2, see online supplementary material) and cloned into pGBKT7 vector. The pGBKT7-*MYB44* and pGADT7-*ABI5* were co-transformed into Y2H Gold yeast cells and grew on the selection medium (SD/−Trp-Leu-Ade-His) to verify protein interaction [58].

### BiFC assays

For BiFC analysis, MYB44 was tagged with the C-terminal part of YFP (C-YFP) using the pFGC5941-C-YFP vector, and ABI5 was tagged with the N-terminal part of YFP (N-YFP) using the pFGC5941-N-YFP vector as previously described [59]. The fluorescence signal was detected using a confocal microscope (LSM 800, Carl Zeiss, Germany) after 2 d of inoculation.

### Pull-down assays

Full-length CDS of *ABI5* and *MYB44* were inserted into pET32a and pGEX4T-1, respectively. The fusion proteins of HIS-ABI5 and GST-MYB44 were induced by 0.1 mM IPTG. Empty plasmid was used as control. GST pull-down assays were carried out as previously described [41].

### Firefly luciferase complementation imaging (LCI) assay

For LCI assay, the CDS of *ABI5* was amplified and cloned into the pCAMBIA1301-nLUC vector and *MYB44* was inserted into the pCAMBIA1301-cLUC vector. *A. tumefaciens* harboring the indicated plasmids were co-infiltrated into the leaves of *N. benthamiana*. LCI assay was performed as described previously [60].

### Co-IP assays

*A. tumefaciens* strain GV3101 carrying the pFGC5941-MYC-*ABI5* and pFGC1008-*MYB44*-FLAG plasmids were infiltrated into the *N. benthamiana* leaves. After inoculation for 2 d, total proteins were isolated with isolation buffer [50 mM Tris–HCl (pH 8.0), 1 mM EDTA, 150 mM NaCl, 0.2% Triton X-100 (Solarbio, T8200), 1 mM phenylmethylsulphonyl fluoride (Solarbio, P8340), 1 protease inhibitor cocktail tablet (MedChemExpress, HY-K0011)], and were centrifuged at 12000 r at 4°C for 15 min. The proteins were immunoprecipitated with anti-FLAG magnetic beads (MedChemExpress, HY-K0207) according to the manufacture’s instruction. For analysis the stability of MYB44, the *N. benthamiana* leaves were injected with 0.1 mM MG132 for 2 h, and then the leaves were harvested.

### Immunoblotting analysis

Immunoblotting analysis was conducted as previously described [54]. The proteins were denatured and separated using 12% SDS-PAGE, and transferred to a PVDF membrane (Millipore, IPVH00010). The membrane was blocked with 5% skim milk powder at 25°C for 1 h, and then probed with a mouse anti-GST antibody (Abmart, M20007), mouse anti-HIS antibody (Abmart, M30111), mouse anti-FLAG antibody (Abmart, M20008), mouse anti-ubiquitin monoclonal antibody (Sigma-Aldrich, U0508), mouse anti-MYC antibody (Abmart, M20002), or mouse anti-actin monoclonal antibody (Sigma-Aldrich, A0480). At last, the membrane was probed a goat anti-mouse HRP-linked antibody (Abmart, M21001), and were analysed using the FDbio-Dura ECL kit (FD8020, Hangzhou, China).

### Statistical analysis

Three independent replicates were used for each determination and the results are shown in the mean ± SD (*n* = 3). SPSS software was used for statistical analysis at *P* < 0.05 with Tukey’s test.

## Supplementary Material

Web_Material_uhad089

## Data Availability

The data supporting the findings of this study are available within the paper and its supplementary information files.
