# Supplementary material for: ABI5 promotes heat stress-induced chlorophyll degradation by modulating the stability of MYB44 in cucumber
Source: Hortic Res. 2023 May 4;10(6):uhad089. doi: 10.1093/hr/uhad089 (PMC10273075; doi:10.1093/hr/uhad089)
Supplement: Web_Material_uhad089 [file web_material_uhad089.zip › Figure SA.pdf]

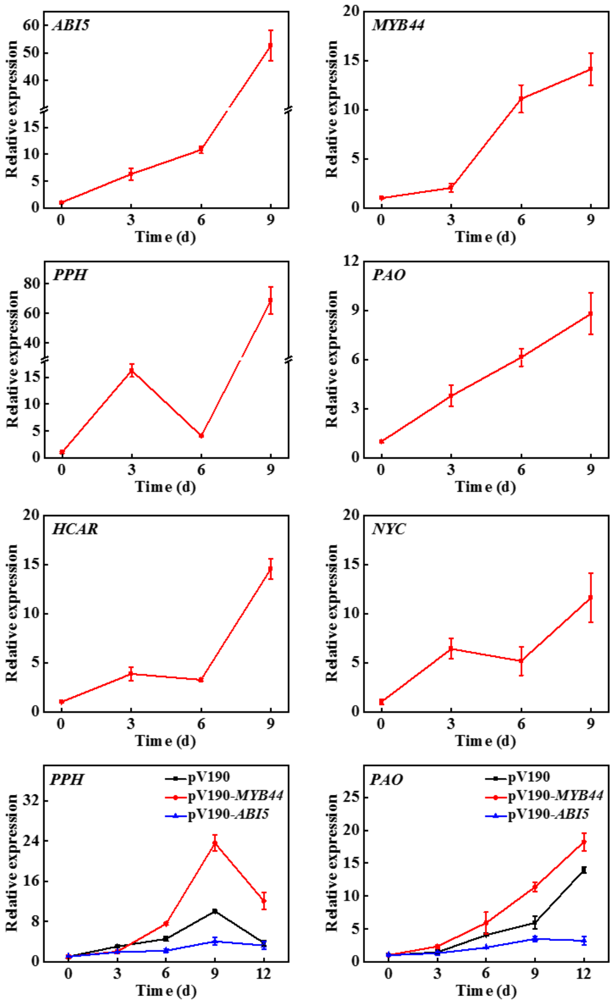

Figure SA. Effects of heat stress on the expression of *ABI5*, *MYB44*, *PPH*, *PAO*, *HCAR*, and *NYC* in cucumber. qPCR analysis of the expression of these genes in cucumber leaves under heat stress for 0, 3, 6, 9, and 12 d. *Ubiquitin* was used as an internal control. The results represent the mean ± SD (n=3).
