# Supplementary material for: ABI5 promotes heat stress-induced chlorophyll degradation by modulating the stability of MYB44 in cucumber
Source: Hortic Res. 2023 May 4;10(6):uhad089. doi: 10.1093/hr/uhad089 (PMC10273075; doi:10.1093/hr/uhad089)
Supplement: Web_Material_uhad089 [file web_material_uhad089.zip › Supplementary Figures - 20230330.pdf]

Figure S1

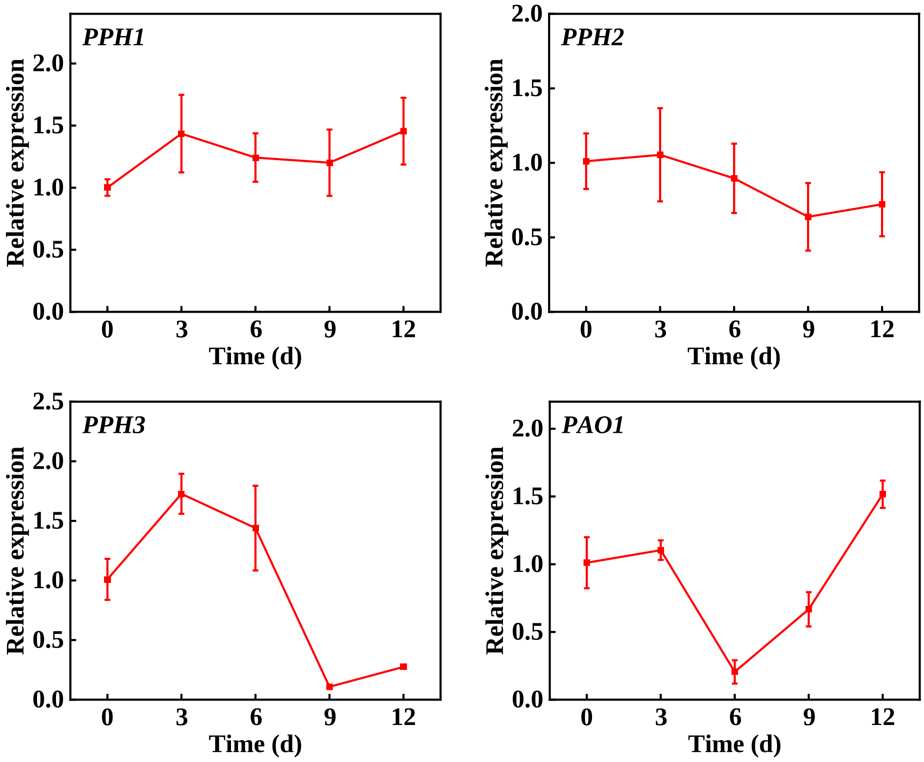

Figure S1. Effects of heat stress on the expression of *PPH* and *PAO* homologous genes. qPCR analysis of the expression of *PPH* and *PAO* homologous genes in cucumber leaves under heat stress for 0, 3, 6, 9, and 12 d. The results represent the mean ± SD (n=3).

Figure S2

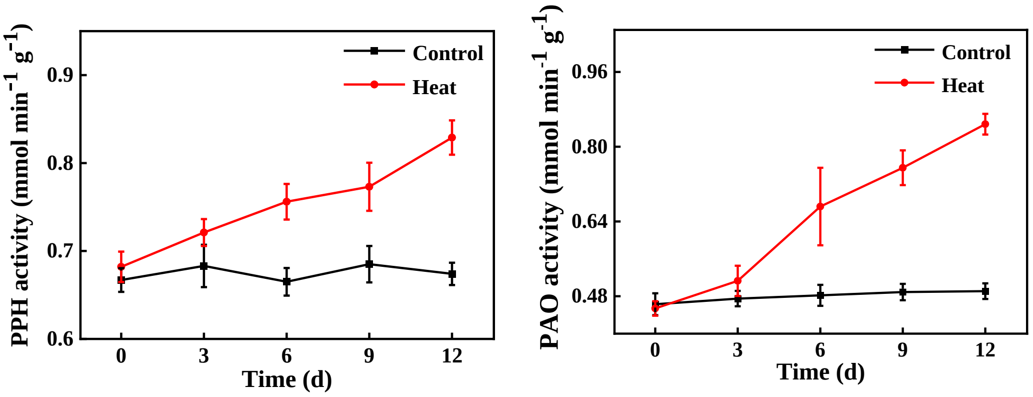

Figure S2. Heat stress enhanced the activity of PPH and PAO. Cucumber plants exposed to heat stress for 0, 3, 6, 9, and 12 d, and the leaves were harvested for analysis the activities of PPH and PAO. The results represent the mean ± SD (n=3).

Figure S3

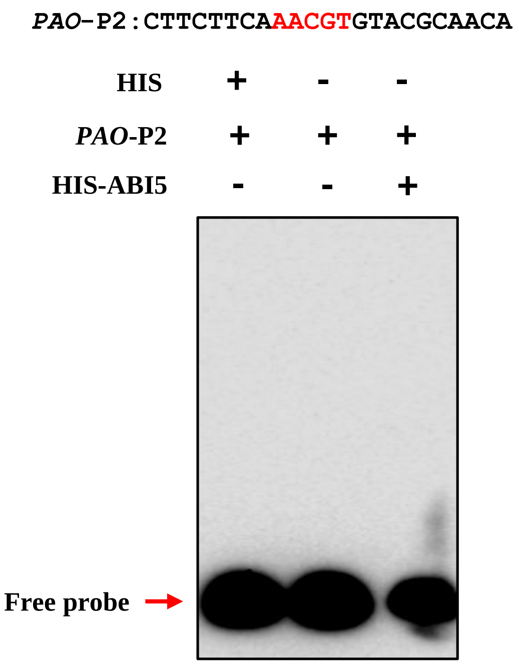

Figure S3. EMSA analysis the binding of ABI5 to the promoter of *PAO*. The purified recombinant HIS-ABI5 protein was mixed with biotin-labeled probes, and the protein-DNA complexes were separated on native polyacrylamide gels. HIS was included as the negative control. Symbols − and + represent absence or presence, respectively.

Figure S4

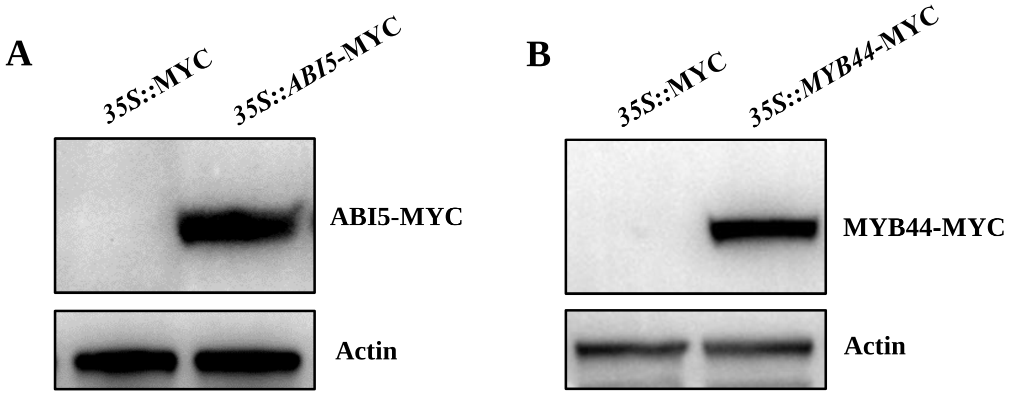

Figure S4. Immunoblotting analysis the expression of MYC-tagged ABI5 and MYB44 in transiently expressed cucumber cotyledons. (A) Immunoblotting analysis the abundance of MYC-tagged ABI5. (B) Immunoblotting analysis the abundance of MYC-tagged MYB44. *Agrobacterium tumefaciens* carrying the indicated vector was injected into the 8-d old cucumber cotyledons, and the injected cotyledons were collected for immunoblotting analysis. Actin was used as the loading control.

Figure S5

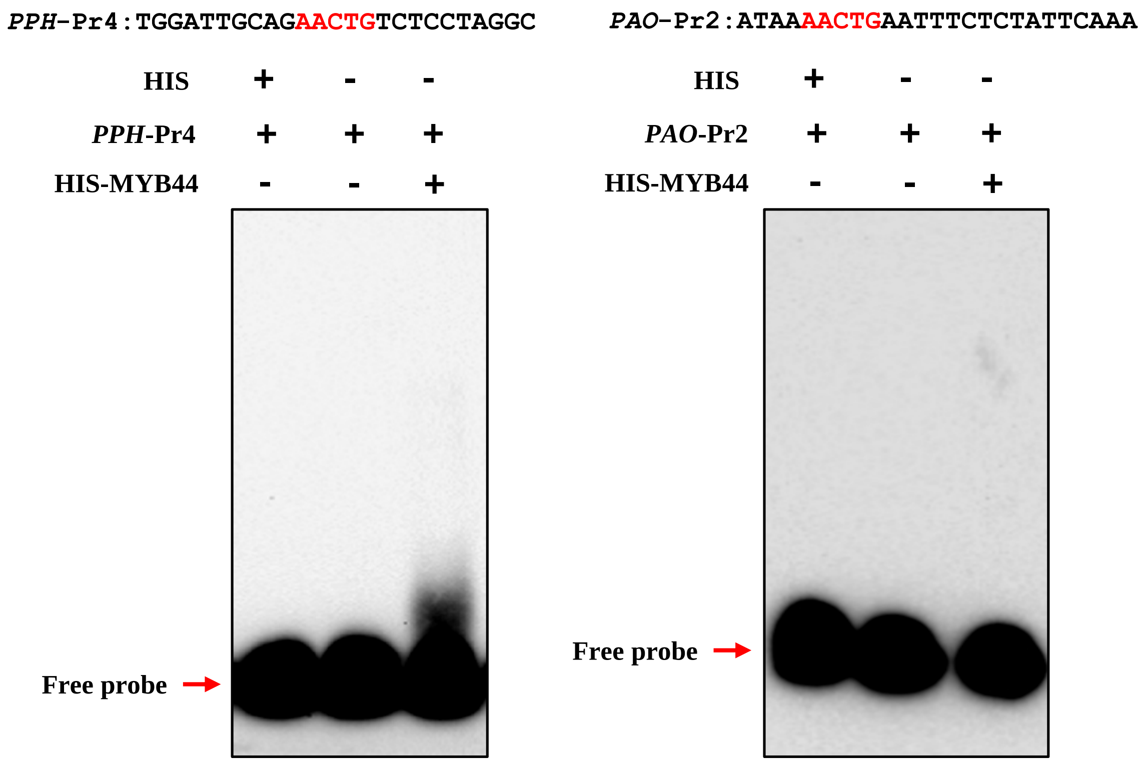

Figure S5. EMSA analysis the binding of MYB44 to the promoter of *PPH* and *PAO*. The purified recombinant HIS-MYB44 protein was mixed with biotin-labeled probes, and the protein-DNA complexes were separated on native polyacrylamide gels. HIS was included as the negative control. Symbols − and + represent absence or presence, respectively.

Figure S6

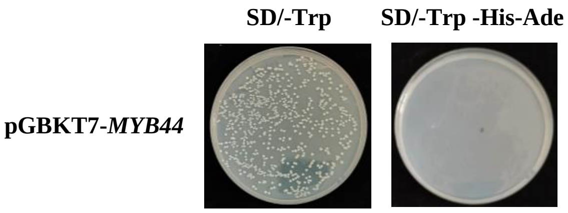

Figure S6. Transcriptional activation analysis of cucumber MYB44 in yeast. The recombinant pGBKT7-*MYB44* vector was transferred into yeast and analyzed by growing on SD/-Trp and SD/-Trp-His-Ade medium.

Figure S7

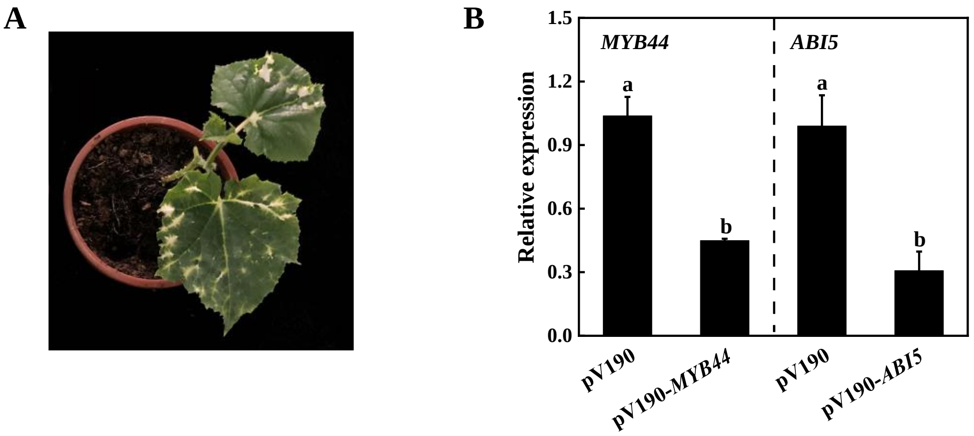

Figure S7. The phenotype of *PDS*-silenced plants and the expression of *PPH* and *PAO* in virus-induced gene silencing (VIGS) plants. (A) The photobleaching phenotype of *PDS*-silenced plants. (B) The expression of *PPH* and *PAO* genes was analyzed after *Agrobacterium*-infection for 30 d in VIGS plants. Results are presented as the means of 3 biological replicates (± SD). Different letters on each column indicated significant differences at $P < 0.05$.

Figure S8

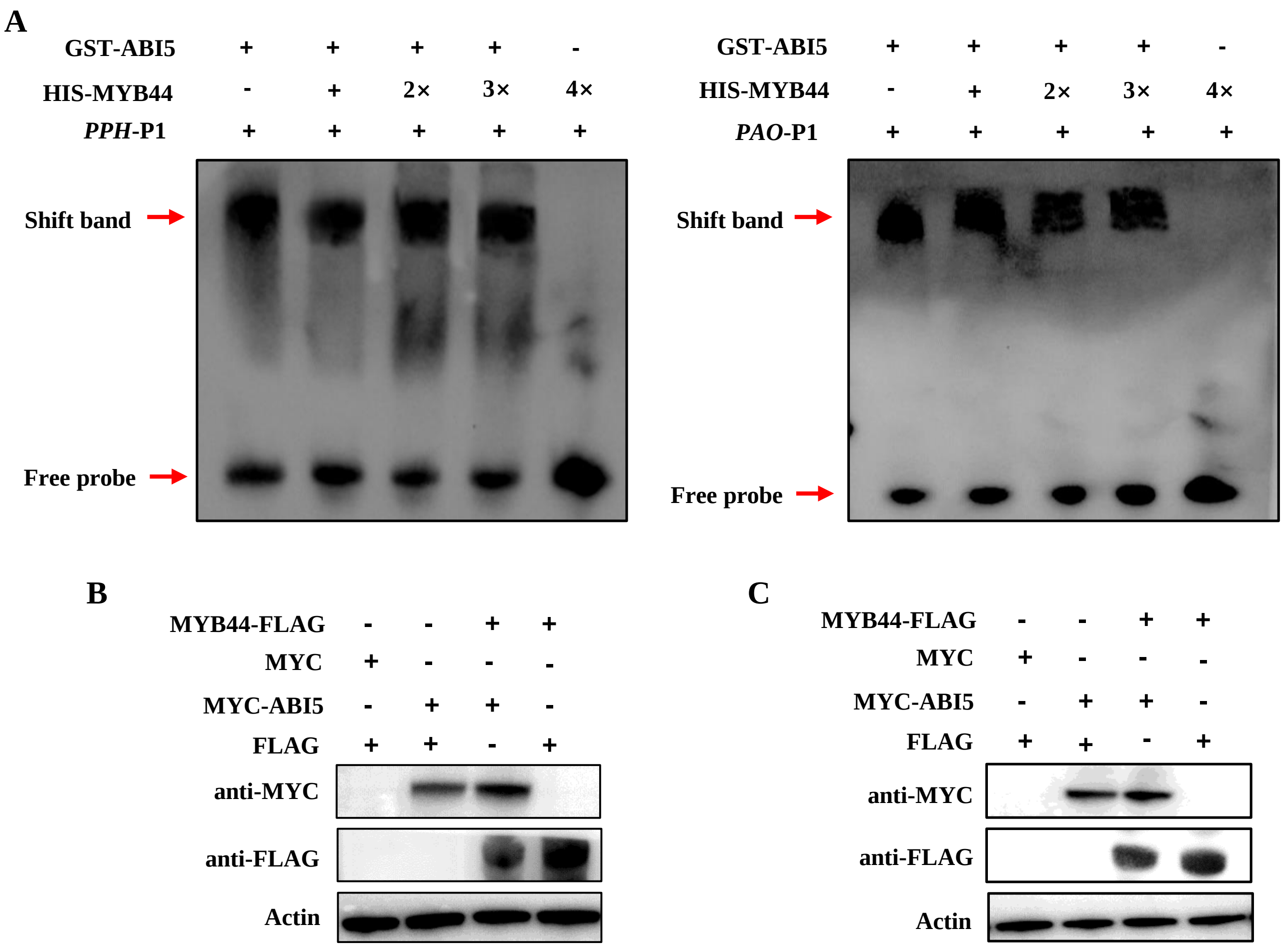

Figure S8. MYB44 does not interfere the binding of ABI5 to the promoters of *PPH* and *PAO*, and western blot analysis the protein levels of ABI5 and MYB44 in the luciferase assay. (A) Electrophoretic mobility shift assay (EMSA) showed that increasing amounts of HIS-MYB44 protein did not affect the binding of GST-ABI5 to the promoters of *PPH* and *PAO*. (B) The protein levels of ABI5 and MYB44 in the luciferase assay analysis the expression of *PPH*. (C) The protein levels of ABI5 and MYB44 in the luciferase assay analysis the expression of *PAO*. Actin was used as the loading control.
