# Supplementary material for: ABI5 promotes heat stress-induced chlorophyll degradation by modulating the stability of MYB44 in cucumber
Source: Hortic Res. 2023 May 4;10(6):uhad089. doi: 10.1093/hr/uhad089 (PMC10273075; doi:10.1093/hr/uhad089)
Supplement: Web_Material_uhad089 [file web_material_uhad089.zip › Supplementary Tables-20230330.pdf]

Table S1. Primers used for qPCR assays in cucumber.

Gene name	Accession number	Forward primer	Reverse primer
PPH	CsaV3_7G030660	5'-GTTATCGTTCCCA GTTTA-3'	5'-AAGTATTGGTGCT GAGTTA-3'
PAO	CsaV3_1G014120	5'-ACCCTAATTCACCT ACCC-3'	5'-TTTCATCTATTCTCC CTTC-3'
MYB44	CsaV3_6G043570	5'-GGATGGCTTCTTTA GTGC-3'	5'-TGTAAGTAGGCTTTGT TGAT-3'
ABI5	CsaV3_3G037220	5'-TAAACAACGCTGG GACAA-3'	5'-GGATGGCTTCTTTAG TGC-3'
HCAR	CsaV3_3G011480	5'-GTGAAGGCAGATG ACGAT-3'	5'-AGTGAATAACGAGCG AAC-3'
NYC	CsaV3_1G024260	5'-GCAGACAGAATCCG AAAC-3'	5'-ATGATGAAGGCACAA ACG-3'
PPH1	CsaV3_4G005750	5'-GGGTTTACTGCCTTG GTT-3'	5'-GGTTTCTTCTGGCTCT TC-3'
PPH2	CsaV3_4G017470	5'-ACTCGGACAAGCCA AATC-3'	5'-GAACAATGCCTTTACA GAT-3'
PPH3	CsaV3_5G005460	5'-AGGGTTCTTATTATC CAGG-3'	5'-TTCCCACTATCCATTC AC-3'
PAO1	CsaV3_1G014100	5'-TGACACAAAGCTTCC TTTACT-3'	5'-AGTAAAGGAA GCTTTGTGTCA-3'
Actin	CsaV3_6G041900	5'-CAGGAATCCACGAA ACTACT-3'	5'-AGACCCTCCAATCCAAA CAC-3'
Ubiquitin	CsaV3_2G007660	5'-CCTTATTGACCAAC CAGTAGT-3'	5'-GGACAATGTTGATTTCC TCG-3'

Table S2. Primer sequences for vector construction.

Primer name	Forward primer	Reverse primer
pET32a-*MYB44*	5'-gctgatatcggatccgaattcATGGCGCTTA CCCGTAAAGA-3'	5'-tgcggccgcaagcttgtcgacTTAAAA GCTAACGTTCTTAATCCCACC-3'
pET32a-*ABI5*	5'-gctgatatcggatccgaattcATGGTTGT AAAAGAATCAGACATGATT-3'	5'-tgcggccgcaagcttgtcgacTTACTGAA TAACTATCAAAGCTGTACTAGAAA-3'
pGEX4T-1-*MYB44*	5'-gatctggttccgcgtggatccATGGCGCTT ACCCGTAAAGA -3'	5'-ctcgagtcgacccgggaattcTTAAAAGCTAA CGTTCTTAATCCCAC-3'
pGEX4T-1-*ABI5*	5'-gatctggttccgcgtggatccATGGTTG TAAAAGAATCAGACATGATT-3'	5'-ctcgagtcgacccgggaattcTTACTGAAT AACTATCAAAGCTGTACTAGAAA-3'
C-YFP-*MYB44*	5'-GCtctagaATGGCGCTTACCCGT AAAGA-3'	5'-CGCggatccAAAGCTAACGTTCTTA ATCCCACC-3'
N-YFP-*ABI5*	5'-acgatgacgacaagcatttaaatATGGTTGT AAAAGAATCAGACATGATT-3'	5'-gatggatcttctagaggatccCTGAATAACT ATCAAAGCTGTACTAG-3'
pCAMBIA-cLUC-*MYB44*	5'-tacgcgtcccggggcggtaccATGGCGCT TACCCGTAAAGA-3'	5'-cagtcgacgcgttgtggatccTTAAAAGCTAA CGTTCTTAATCCCACC-3'
pCAMBIA-nLUC-*ABI5*	5'acgggggacgagctcggtaccATGGTTGT AAAAGAATCAGACATGATT-3'	5'-cagtcgacgcgttgtggatccCTGAATAACTA TCAAAGCTGTACTAGAAAAA-3'
pGKBT7-*MYB44*	5'-atggccatggaggccgaattcATGGC GCTTACCCGTAAAGA-3'	5'-gcaggtcgacggatccccgggTTAAAAGCT AACGTTCTTAATCCCAC-3'
pGADT7-*ABI5*	5'-gccatggaggccagtgaattcATGGTTGT AAAAGAATCAGACATGATT-3'	5'-cagctcgagctcgatggatccCTGAATAACT ATCAAAGCTGTACTAGAAAAA-3'
pAbAi-*proPPH*	5'-cttgaattcgagctcggtaccAAGAACTAA ATTATCATAAAGTGAAAAAACC-3'	5'-atacagagcacatgcctcgagATTAATATT TGGGGATTGTCAAAGAA-3'
pAbAi-*proPAO*	5'-cttgaattcgagctcggtaccAGTTAATATT TATCTTAGTCATGCTTAAATCC-3'	5'-atacagagcacatgcctcgagGAGATGTTGC GTACACGTTTGAA-3'
pGreenII0800-*proPPH*	5'-ctatagggcgaattgggtaccAAGAACTAA ATTATCATAAAGTGAAAAAACC-3'	5'-atcgataccgtcgacctcgagATTAATATTTG GGGATTGTCAAAGAA-3'
pGreenII0800-*proPAO*	5'-ctatagggcgaattgggtaccAGTTAATATT TATCTTAGTCATGCTTAAATCC-3'	5'-atcgataccgtcgacctcgagGAGATGTTGCG TACACGTTTGAA-3'
pFGC1008-*MYB44*-FLAG	5'-ttacaattaccatggggcgcgccATGGCGC TTACCCGTAAAGA-3'	5'-gtccttgtagtcagaggtaccAAAGCTAACGT TCTTAATCCCACC-3'
pFGC5941-MYC-*ABI5*	5'-catatggggctgcaggaattcATGGTTG TAAAAGAATCAGACATGATT-3'	5'-gggactagaactagtggatccTTACTGA ATAACTATCAAAGCTGTACTAG-3'
pV190-*MYB44*	5'-aggactttacttaatggatccTTGATGATG AATGAGGGATATGAAG-3'	5'-cctagacctataactggatccTAGAGGTACC TGCGCCGTTG-3'
pV190-*ABI5*	5'-aggactttacttaatggatccATGGTTGTAA AAGAATCAGACATGATT-3'	5'-cctagacctataactggatccCACCGGCAAGT GACCCTGA-3'
pV190-*PDS*	5'-aggactttacttaatggatccCAGGAGAAG CATGGCTCTAAGATG-3'	5'-cctagacctataactggatccTGGAACTCCA ACTAATTTTTCCAGC-3'
pCAMBIA1300-*MYB44*-MYC	5'-atacaccaaatcgactctagaATGGCGCT TACCCGTAAAGA-3'	5'- gagcttttgctccatggtaccAAAGCTAACG TTCTTAATCCCACC-3'
pCAMBIA1300-*ABI5*-MYC	5'- atacaccaaatcgactctagaATGGTTGTA AAAGAATCAGACATGATT-3'	5'- gagcttttgctccatggtaccCTGAATAACTAT CAAAGCTGTACTAGAAAAAT-3'

Table S3. Primers used for ChIP-qPCR.

Gene name	Forward primer	Reverse primer
PPH-MYB44	5'-AGCTTAAACAACAGTTACCCAA-3'	5'-GCCTAGGAGACAGTTCTGC-3'
PPH-ABI5	5'-AATTTCTCCCACGTTACGGCT-3'	5'-GAAATTGGGAAGAGTTAA-3'
PAO-MYB44	5'-ATAAAACTGAATTTCTCTATTCA-3'	5'-CAAAGGGTTCAAACAGGG-3'
PAO-ABI5	5'-CTCATTTGTCACGTTTTCTCA-3'	5'-CAAAGGGTTCAAACAGGG-3'
